# Deep neck space infections: an upward trend and changing characteristics

**DOI:** 10.1007/s00405-019-05742-9

**Published:** 2019-12-03

**Authors:** Jarno Velhonoja, Meira Lääveri, Tero Soukka, Heikki Irjala, Ilpo Kinnunen

**Affiliations:** 1 Department of Otorhinolaryngology and Head and Neck Surgery, Turku University Hospital and University of Turku, Kiinanmyllynkatu 4-8, 20520 Turku, Finland; 2 Department of Oral and Maxillofacial Diseases, Turku University Hospital and University of Turku, Lemminkäisenkatu 2, 20520 Turku, Finland

**Keywords:** Deep neck space infections, Abscess, Complications, Hyperbaric oxygen therapy, Gas

## Abstract

**Purpose:**

This study reviews our experience with deep neck space infections (DNIs) requiring surgical intervention, including cervical necrotizing fasciitis. The aim of the study was to identify predisposing and aggravating factors of the disease and recognize the possible factors that can lead to life-threatening complications and slow down the healing process.

**Methods:**

We compare the results to previous data from 1985 to 2005 to find possible alterations and changing trends. The characteristics of four lethal cases are described. This retrospective analysis includes patient data from 2004 to 2015 in tertiary referral hospital and in total, 277 patients were found.

**Results:**

Surgical drainage through a neck opening ± intraoral incision was made in 215 (77.6%) patients, an intraoral incision was only made in 62 patients (22.4%). ICU care was needed in 66 (23.8%) cases. Odontogenic etiology (44.8%) was the most common origin. The most common comorbidity was a psychiatric disorder and/or dementia and occurred in 55 (19.9%) patients. Patients with underlying illnesses were more likely to be admitted to the ICU (*p* = 0.020), required a longer ICU stay (*p* = 0.004) and repeated surgery (*p* = 0.009). Gas formation seemed to be predictive of a more severe course of infection. Early extraction of the odontogenic foci was related to a lower length of stay (LOS) (*p* = 0.039).

**Conclusion:**

The annual numbers have risen from 14 to 24 cases per year when compared to previous data. DNIs remain a cause of lethal complications; the mortality was 1.4% and overall complications occurred in 61 (22.0%) patients.

## Introduction

Deep neck space infections (DNIs) are a group of severe bacterial infections in potential spaces and fascial planes of the neck. Abscesses, cellulitis and phlegmons can spread along these fascial planes from the skull base to the mediastinum and cause serious and potentially life-threatening complications [1].

Some authors have stated that DNIs are currently less frequent than in the past [2, 3]. Nevertheless, the DNIs remain a continuous challenge because of the significant morbidity and mortality rates [4–8]. From Finland there is evidence that DNIs have become more prevalent in recent decades [9]. The most common etiology is odontogenic (35–42%) and pharyngotonsillar infections. Other causes include salivary gland infections, penetrating or blunt trauma, a foreign body, iatrogenic factors such as prior surgery and dental procedures, neoplasm, lymphadenitis and infected cysts. Unknown etiology varies in the literature and remains around 8–57% of cases [5, 10–12]. DNIs require prompt and accurate management. This includes managing the often-compromised airway, adequate antimicrobial therapy, surgical incision and drainage of the abscess, identifying and treating the possible cause (removal of the causal teeth or tonsils) and treatment of complications. Compromised airway, descending mediastinitis, thrombosis of the internal jugular vein, arterial erosion, pneumonia, meningitis and intra-cranial extensions are the potentially lethal complications, especially to immunocompromised patients or patients with comorbidities [7, 11].

The aim of the study is to identify the causes and predisposing factors of DNIs and recognize the possible factors that can lead to severe complications and slow down the healing process. We also demonstrate the changing trends in morbidity, mortality and admissions for DNIs in our tertiary referral hospital compared to previously published data from 1985–2005.

## Materials and methods

This study is a retrospective analysis of clinical data collected from medical records between 1.1.2004 and 30.11.2015 at Turku University Hospital. A wide range of ICD10 codes was used to distinguish all the patients with deep neck infection (including J39.0, L02.1, K11.3, J39.1, J39.0, J36, A48.0, M72.6, K12.2, K02.1, K04.1, K04.4, K04.5, K04.60–K04.63, K04.69, K04.7, K05.5, K05.22 and K05.32). Of the 2588 patients identified 277 met the inclusion criteria, defined as a DNI requiring surgical treatment, including intraoral or extraoral openings, and admission to a specialized ward or ICU. We excluded peritonsillar abscesses without any complications and the DNI patients, who were treated conservatively.

A review is presented of patient demographics, associated systemic diseases, airway status, treatment, operations, re-operations, duration of hospitalization, intensive care unit (ICU) days, bacterial cultures, hyperbaric oxygen therapy (HBO), complications, and the outcomes for DNIs.

IBM SPSS Statistics version 24 was used to analyze data. A Pearson Chi-Square test was used to compare categorical data between groups with an asymptotic 2-sided significance *p* value below 0.05 being considered significant. A Mann–Whitney *U* test was utilized to compare continuous variables between the groups. A two-sided *p* value of less than 0.05 was considered statistically significant. One sample *t* test was used to compare means and a Fisher’s exact test when dealing with a group size under five.

## Results

### Demographic and clinical data

Turku University Hospital serves as a tertiary referral hospital for Southwest Finland and Satakunta district, with a population of 870,000 people. Because Turku University Hospital is the national center for HBO treatment, some patients (*n* = 12) were also referred from other central hospitals in Finland. In total *n* = 277 patients operated for DNIs were included in this study. There was a clear male predominance, 179 (64.6%) patients were male and 98 (35.4%) female. Age distribution was 0.5–92 years (mean 42.3; SD 20.5) and there was no difference in age between males (42.0; SD 18.3) and females (42.9; SD 18.3). Male gender was associated with poor dental status which was noted in 76 (75.2%) males versus 25 females (25.5%; *p* = 0.005) as well as the odontogenic etiology (49.7%; *p* = 0.016). Oral health evaluation was based on status findings and/or dental imaging noted in patient records.

### Comorbidities

Associated systemic disorders are specified in Table 1. 114 (41.2%) patients had some or several comorbidities. Comorbidities between genders were equally distributed (*p* = 0.996). Psychiatric disorders and/or dementia was found in 55 (19.9%). 31 (11.2%) were diabetic patients on medication of which 17 (6.1%) patients had insulin treatment. 23 (8.3%) were classified as immunocompromised including patients receiving medications for autoimmune diseases or other known immunodeficiency. Alcohol abuse was mentioned in the patient history of 29 (10.5%) patients. Heart and vascular diseases were reported in 20 (7.2%) of patients. These included coronary artery disease or previous myocardial infraction, congestive heart failure, previous stroke or transient ischemic attack (TIA), however, solitary hypertension was excluded. 11 (4.0%) had malignancy in their patient history. Eight (2.9%) had clinically important liver disease, including hepatitis and cirrhosis. Six (2.2%) patients had a history of intravenous substance abuse.

**Table 1:** Comorbidities of patients with deep neck infections

Comorbidity	No. patients (*n* = 277)	%
Psychiatric diagnosis or dementia	55	19.9
Diabetes	31	11.2
Alcohol abuse	29	10.5
Immunocompromised	23	8.3
Heart and vascular disease	20	7.2
Malignancy (inc. previous)	11	4.0
Liver disease	8	2.9
I.v. substance abuse	6	2.2
One or several comorbidity	114	41.2
Healthy	163	58.8

Poor dental status was noted in 101 (36%) of all patients based on status findings and/or dental imaging but was as high as 50.1% (*n* = 28) in the psychiatric/dementia group. The difference was significant (*p* = 0.013). Odontogenic etiology in the psychiatric/dementia group was increased (54.5%), however, it was statistically not significant (*p* = 0.091). As expected in the whole odontogenic subgroup poor dental status was noted in 73 (59%) cases (*p* = 0.000).

### Etiology

An odontogenic origin was the most common reason for DNI accounting for 124 (44.8%) of the cases. Twenty (16.1% of the odontogenic) patients had dental surgery before admission to the hospital. The second common etiology was of a pharyngeal or tonsillar origin 104 (37.5%). The other sources were lymphadenitis in 12 (4.3%) and sialadenitis, neoplasm, infected cyst accounting for four cases (1.4%) each. Two (0.7%) penetrating trauma to the cervical region, an otogenic infection (0.4%), a foreign body (0.4%) and a post-operative infection (0.4%) were identified. The origin of the infection was not determined in 20 (7.2%) cases.

### Diagnosis and treatment

**Fig. 1:**
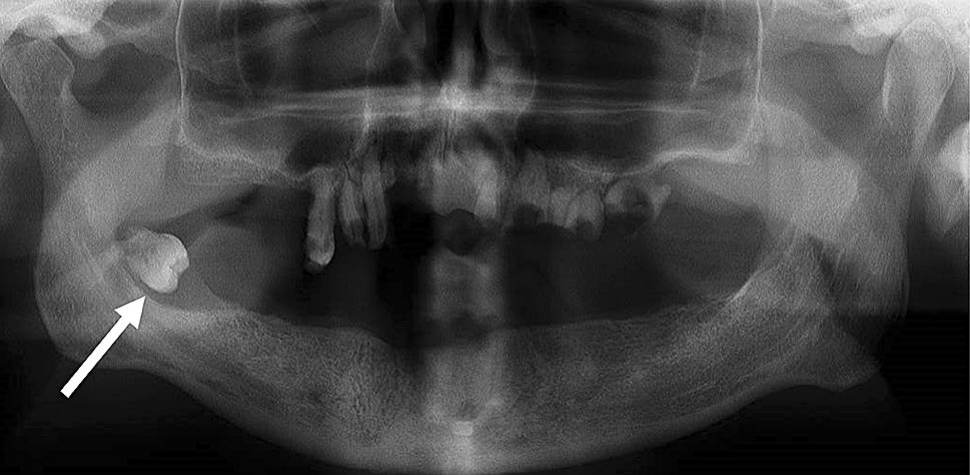
Orthopantomography showing dental foci and a wide radiolucency around the unerupted D48 (arrow)

**Fig. 2:**
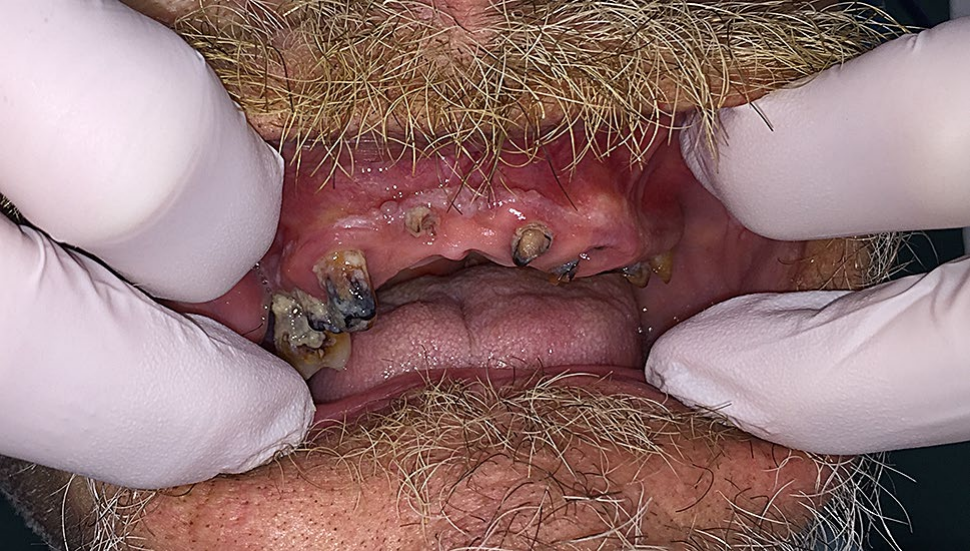
Intraoperative picture of poor dental status

**Fig. 3:**
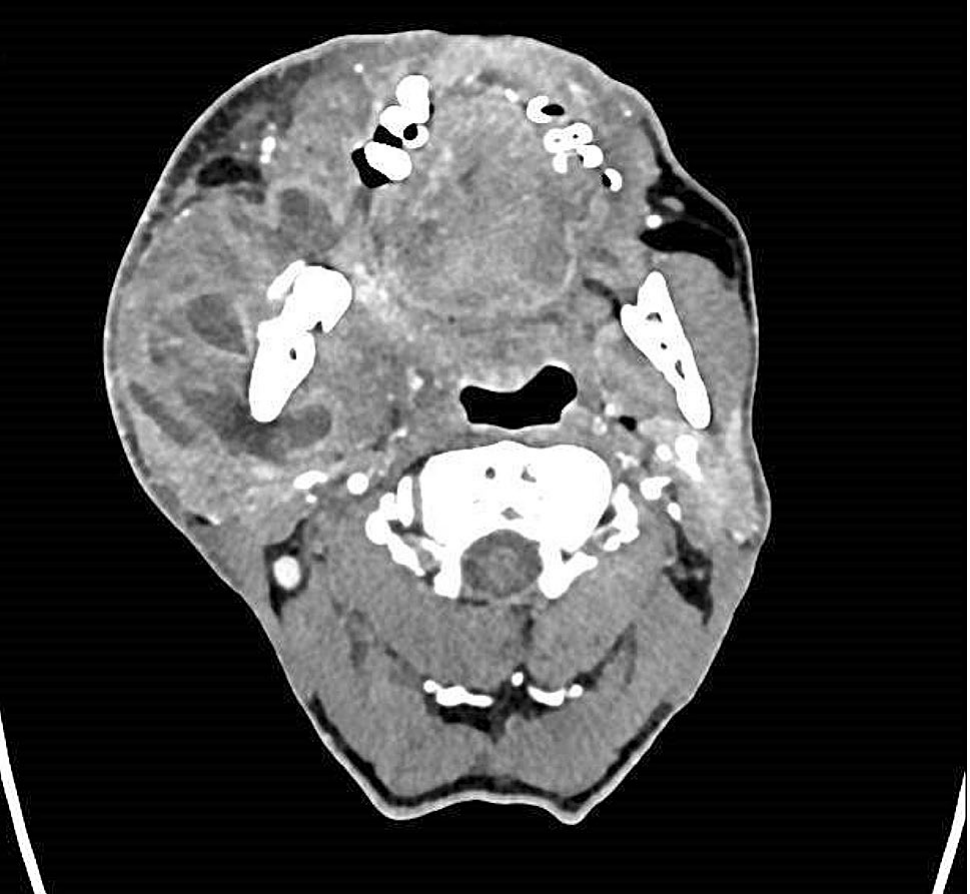
Contrast-enhanced CT scan presenting a multilocular abscess of a middle-aged male patient who has a history of epilepsy and smoking, presented with a few days neck and cheek swelling, trismus and C-reactive-protein reported 305 mg/L at admission

Diagnosis of DNI was based upon a thorough otorhinolaryngological examination usually together with fiberoptic nasopharyngoscopy and imaging. In cases of an odontogenic foci, an oral and maxillofacial surgeon was consulted. Orthopantomography (OPG) was carried out on 147 (53.1%) patients. For illustration, in Figs. 1 and 2 odontogenic foci and poor dental status of the DNI patient can be recognized. The most common imaging technique was a contrast-enhanced computer tomography (CECT) of the neck, which was conducted on 174 patients (62.8%). A typical ring-enhancing multilocular abscess of the same patient is demonstrated in Fig. 3. The initial CECT included the mediastinum when a descending infection was suspected. Magnetic resonance imaging was obtained from 37 (13.4%) patients and five patients underwent both imaging examinations. Ultrasonography was used on 11 (4.0%) patients. 37 (13.4%) patients were treated only with an OPG and seven patients (2.5%) were treated using no imaging examinations; in these cases, diagnosis of DNI was made by a clinical examination and a surgical exploration.

Airway management is crucial when treating deep neck space infections. 36 patients (13.0%) needed a tracheostomy. A wide spectrum of intravenous empirical antimicrobial therapy was administered to all patients and it was later specified according to microbiological findings and drug sensitivity tests.

**Fig. 4:**
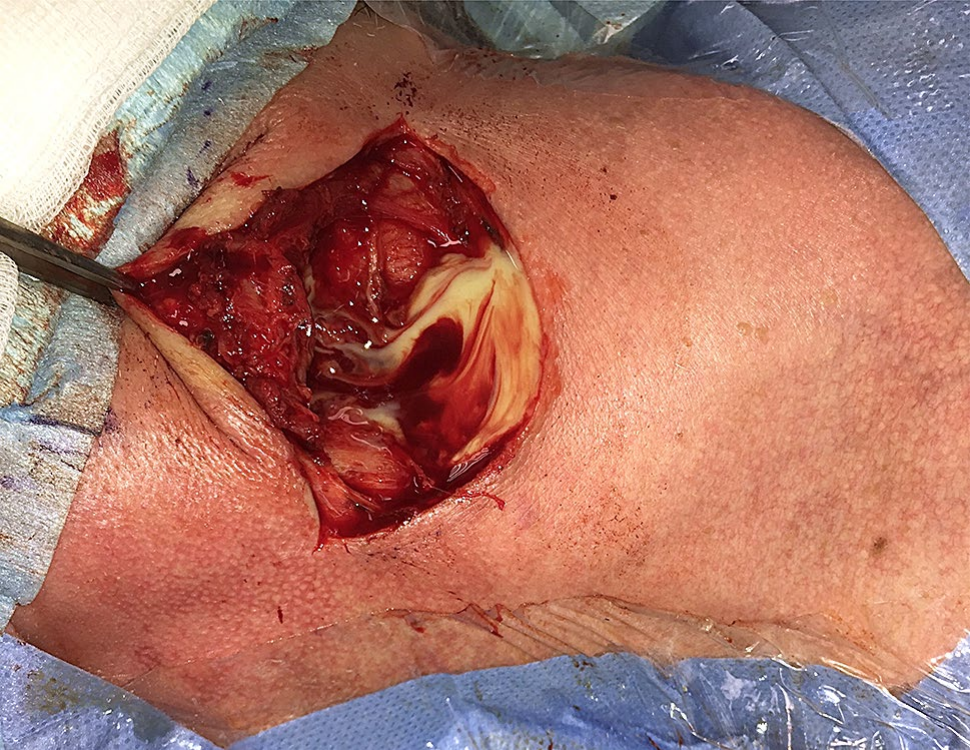
A neck incision and drainage was performed immediately on admission with a simultaneous extraction of the all remaining dentation. After a 4-days course of intravenous antibiotics, the patient was released from the hospital to outpatient follow-up without complications

Cervical incision (Fig. 4**)** and surgical drainage ± intraoral incision and removal of necrotic tissue was made in 215 (77.6%) patients and an intraoral incision in 62 (22.4%). Tonsillectomy was performed in 63 cases (22.7%). Dental extraction was made in 93 (33.6%) cases at the referral hospital and overall to 113 (40.8%) of the patients and to 100 patients (80.6%) (*p* < 0.000) in the odontogenic subgroup. It is notable that the length of stay (LOS) was significantly reduced from a median of 7 days to 5 days (*p* = 0.039) when extraction was made simultaneously (same day) with the incision and drainage compared to those with delayed extraction (range 1–9 days). However, the differences in complications (*p* = 0.153), the need for ICU (*p* = 0.105) or ICU stay days (*p* = 0.223) were not distinguished between early and late extraction.

47 patients (17.0%) had revision surgery through neck opening(s) and seven (2.5%) patients also needed mediastinal canalization or thoracotomy. In the subgroup analysis, the odontogenic group was more likely to need repeated surgery (*p* = 0.022) as well as the patients with comorbidity (*p* = 0.009). The mean overall LOS (*n* = 260) in our tertiary referral center was 8.5 days, median 6.00 days (range 2–114 days, SD 10.1). The data of 17 (6.1%) patients were missing because of their transfer to another hospital for follow-up treatment. 66 (23.8%) patients were treated in the intensive care unit. The ICU stay of four patients was unknown because of their transfer to another hospital. The mean ICU stay (*n* = 62) was 7.2 days, median 5 days (range 1–36 days, SD 7.4). Patients with underlying illnesses were more likely to be admitted to the ICU (*p* = 0.020) and had a longer ICU stay, their median days being 2 vs. 7 (*p* = 0.004). Diabetic patients had an overall longer hospital stay with a median of eight days (range 2–48 days, SD 13.0) and a stay of over 6 days (all patients’ median) occurred more frequently. There was a significant difference between diabetic and non-diabetic patients (*p* = 0.040). Similarly, ICU treatment was needed more frequently for diabetic patients (*n* = 16; 51.6%; p < 0.000). HBO therapy was mainly used once a day (except twice for two patients) as an adjuvant therapy for 42 (15.2%) patients during the intensive care period with a median of 6 days (range 1–15 days). HBO treatment was begun immediately (at least within 24 h) after initial surgery in 23 (54.8%; range 0–12 days) of the cases.

### Complications

Complications occurred in 61 (22.0%) patients and are specified in Table 2. 18 (6.5%) patients developed necrotizing fasciitis (NF) which is a rapidly progressing fulminant complication. NF was defined as a severe soft tissue infection (need for ICU treatment) with intense pain being mentioned or gas between the fascial layers; in all cases clinically confirmed necrotic tissue was found during surgery. Descending mediastinitis was diagnosed in 12 (4.3%) patients and 7 patients needed operations involving mediastinum. Two patients needed repeated thoracic surgery in addition to neck revisions to control the infection. However, all except one of the patients who arrived in an exceedingly advanced stage, suffering from mediastinitis, survived and were discharged from the hospital. HBO treatment was used in nine mediastinitis patients (75.0%). Of all the DNI patients, 18 (6.5%) had multiple complications, nevertheless most of the patients 216 (78.0%) recovered without aggravating factors. The mortality rate was 1.4%. Comorbidities were associated with increased mortality (*p* = 0.015). There were four lethal outcomes, which are categorized in Table 3. In these cases, infection was primarily advanced and involved multiple spaces. Three of the cases were categorized as NF. Two were of odontogenic origin and two pharyngotonsillar.

**Table 2:** Complications of deep neck space infections

Complication^a^	No. patients (*n* = 277)	%
Necrotizing fasciitis	18	6.5
Pneumonia	16	5.8
Sepsis (blood culture positive)	13	4.7
Mediastinitis	12	4.3
Neural damage	12	4.3
Internal jugular vein thrombosis	6	2.2
Total airway obstruction	4	1.4
Death	4	1.4
Orocutaneus fistula	3	1.1
Disseminated intravascular coagulation (DIC)	2	0.7
Iatrogenic tracheal stenosis	2	0.7
Delirium	1	0.4
Pulmonary embolism	1	0.4

^a^18 (6.5%) patients had multiple complications

**Table 3:** Clinical characteristics of lethal outcomes

Case no	Gender/age	Comorbidity	Etiology	Type of infection	Complication	CRP (max)	WBC (max)	Surgery	Bacterial culture	Blood culture
1	F/56	Alcohol abuse, liver disease	Odonto-genic	NF	Int. jug. vein thrombosis, resuscitation, anoxia, mediastinitis	367	18.5	Neck I&D, dental extr. of 6 teeth	*Strep. constellatus, Prevotella species*	No culture
2	F/81	Immuno-compromised, pansytopenic, lung tumor	Periton-sillar abscess	Gas-producing cellulitis	Sepsis, tetraparesis of unknow cause	390	9.9	Neck I&D, tonsillectomy	*Strep. pyogenes, Strep. constellatus, Prevotella melaninogenica*	*Strep. pyogenes*
3	M/56	Insulin diabetic, COPD, psychiatric disorder	Odonto-genic	NF	DIC	33	46	Neck I&D, dental extr.	*Escherichia coli, Klebsiella oxytoca, Prevotella denticola, Strep. anginosus*	No culture
4	F/53	Immuno-compromised, ankylosing spondylitis	Tonsillitis	NF	Sepsis, DIC	327	1	Neck and intraoral I&D	No culture	*Serratia marcescens*

*CRP* C-reactive protein mg/L, *WBC* white blood cell count E9/L, *NF* necrotizing fasciitis, *I&D* incision and drainage, *DIC* disseminated intravascular coagulation

The complication rate among diabetic patients was slightly augmented being 32.3% when compared to non-diabetics (20.7%) but it was statistically insignificant (*p* = 0.144). Patients with a (former) malignancy had complications more frequently (*n* = 8; 72.7%; *p* < 0.000). All patients having comorbidities seemed not to have associations with complications (*p* = 0.131). However, taking merely the somatic underlying illnesses into account (excl. psychiatric and dementia subgroup), there was a significant connection to complications (*p* = 0.048).

Gas formation was noted in 41 (14.8%) patients in the radiological investigations. It was associated with higher a complication rate 46.3% (*p* < 0.000), increased mortality (*p* < 0.000), need for repeated surgery (*p* < 0.000), longer hospitalization (*p* < 0.000), ICU treatment (*p* < 0.000) and increased ICU days (*p* = 0.008). Not all gas producing infections were categorized as NF because they had a more benign course of infection or a lack of clinically confirmed necrosis. Nevertheless, this subgroup of non-NF cases with gas formation (*n* = 26) had a higher complication rate of 42.2% (*p* = 0.002), increased mortality (*p* = 0.003), longer hospitalization (*p* = 0.001), ICU treatment (*p* = 0.014) and ICU days (*p* = 0.036) but the need for revisions did not differ significantly (*p* = 0.084).

### Bacteriology

Bacterial samples from surgery were available for 226 (81.6%) patients. 33 (14.6%) of the 226 had negative cultures and 193 (85.4%) had positive culture results for one or several bacteria. The most frequent bacterial organisms are presented in Table 4. Polymicrobial growth was present in 59.6% (*n* = 115) of the positive cultures. Most common pathogens to involve were *Streptococcus species n* = 123 (63.7%). *Prevotella species* were found in 70 (36.3%) of the positive cultures but interestingly only in one (0.5%) case was it the only pathogen identified. *Streptococcus anginosus* (*n* = 31) and an unspecified anginosus group streptococci (*n* = 7) accounted together for 19.7% of the bacteria, the *Fusobacterium species* 29 (15.0%), *Streptococcus constellatus* 21 (10.9%) and the other viridans group streptococci 25 (13.0%) were the most frequent findings. *Streptococcus anginosus*, *constellatus* and *intermedius* are part of the anginosus group streptocci (formerly known as the Streptococcus milleri group) and were involved altogether in 35.8% (*n* = 69) of the cases. *Staphylococcus aureus* was found in 22 (11.4%) of the cases.

**Table 4:** Ten most common results in positive bacterial cultures

Bacterial cultures	No. cases	%^a^
*Prevotella species*	70	36.3
*Streptococcus anginosus *and unspecified anginosus group^b^	38	19.7
*Fusobacterium species*	29	15.0
Viridans group streptococci^c^	25	13.0
*Staphylococcus aureus*	22	11.4
*Streptococcus constellatus*	21	10.9
*Streptococcus pyogenes*	15	7.8
*Streptococcus betahaemolyticus non A*	14	7.3
*Sreptococcus intermedius*	10	5.2
*Propionibacterium acnes*	8	4.1
Unspecified polymicrobial growth	29	15.0

^a^Percentage of positive cultures (*n* = 193)

^b^Excluding *Strep. constellatus and intermedius* (listed separately)

^c^Including *Strep. salivarius, gordonii, parasanguinis, mitis group, alfahaemolyticus*

### Comparison to previously published data from our institute

In 2008, Aitasalo published a review of 293 patients of DNIs in Turku University Hospital between 1985 and 2005 [13]. To conduct a relevant evaluation, we excluded the years 2004–2005 from our current data.

In total, 293 patients were analyzed in the former study. Comparison and characteristics are presented in Table 5. A 168 (57%) of the patients were male and 125 (43%) female. Age distribution was 4–91 years, mean 38.5 years. Patients were significantly older in the present study (mean 43.3 years; *p* < 0.000). 61 (20%) were admitted to the ICU and 36 (12%) received HBO therapy and both of these values did not differ in comparison to the present study (*p* = 0.342; 0.112).

**Table 5:** Characteristics of deep neck infections in two time periods

	1985–2005	%	2006–2015	%	*p* value
Patients (*n*)	293		239		
Male	168	57.3	152	63.6	0.142^a^
Female	125	42.7	87	36.4	0.142^a^
Patients/year/mean	14		24 (15–32)		
Age distribution (mean)	38.5		43.3		**0.000**^b^
Age distribution (range)	4.0–91		0.5–92		
ICU treatment	61	20.8	58	24.3	0.342^a^
HBO treatment	36	12.3	41	17.2	0.112^a^
Complications	42	14.3	55	23.0	**0.009**^a^
Comorbidity	51	17.4	103	43.1	**0.000**^a^
Mortality	0	0.0	4	1.7	**0.040**^c^
Etiology					
Odontogenic	64	21.8	101	42.3	**0.000**^a^
Prev. dental surgery	32	10.9	16	6.7	0.091^a^
Pharyngotonsillar	69	23.5	92	38.5	**0.000**^a^
Unknown	54	18.4	16	6.7	**0.000**^a^
Space infected					
Parapharyngeal	114	39	46	19.2	**0.000**^a^
Submandibular	82	28	72	30.1	0.588^a^
Retropharyngeal	32	11	30	12.6	0.560^a^
Multilocular	-		37	15.5	

The significant *p* values are shown in bold

^a^Chi-square test

^b^One sample *t* test

^c^Fisher’s exact test

42 (14%) patients had complications. Specific numbers are unavailable but descending mediastinitis and necrotizing fasciitis were mentioned as being amongst the most common complications. A few other complications such as pleurisy, septic shock and aspiration causing pneumonia were considered rare. The overall complication rate was 23.0% in our more recent data (*p* = 0.009). Neural damages and several other complications (Table 2) were not mentioned in this previous report. Mortality was increased from 0 to 1.7% because of four lethal cases occurring during the later study period. Comorbidities were significantly increased in the recent data (*p* < 0.000). When analyzing only somatic disorders (omitting psychiatric and dementia diagnoses) we still found an increased rate of comorbidities 32.2% (*n* = 77; *p* < 0.000).

Odontogenic and pharyngotonsillar etiology were most common in both groups but the proportion had significantly increased in the recent group (*p* < 0.000). The unknown etiology category was lower (*p* < 0.000) in the latest data. Parapharyngeal, submandibular and retropharyngeal were the spaces most commonly involved in both groups. Only parapharyngeal infections were more frequent in the previous group (*p* < 0.000).

## Discussion

We found DNIs to be an increasing burden on the health care system. In this article, we presented an analysis of 277 DNI patients requiring surgical treatment. DNIs can be mostly well managed in adults with intravenous antimicrobial therapy often combined with the timely incision and drainage [1, 6, 11]. A widespread progression can be prevented with an efficient treatment which also includes airway control. Due to the complex anatomy of the neck, surgical treatment tends to be challenging. Moreover, in present study, severe and lethal complications did occur and associated systemic disorders were shown to aggravate the disease.

The male predominance of DNI patients has been recognized in many studies but the underlying cause remains unclear [5, 7, 8, 11, 14]. The distribution of 64.6% males in this study was in concordance with these previous findings. The males in the study population were associated with both poor dental status and odontogenic etiology which in part could explain the increased morbidity.

The median hospitalization (LOS) was 6 days (mean 8.5). A comparable LOS, a median of 7 days was reported by Tapiovaara et al. 2017, in surgically treated DNIs [12]. A mean LOS of 9.5 days was reported by Parhiscar et al. 2001, 13 days by Huang et al. 2004, and 13.1 days by Ridder et al. 2005 [3, 5, 14]. Early extraction combined with incision and drainage accompanied by antibiotics can be considered as the mainstay of treatment when managing odontogenic infections [15, 16]. The presented results show that the overall hospital stay was significantly reduced when extraction was made immediately (same day) as the incision and drainage, compared to those with delayed extraction. Comparable results were reported recently by Heim et al. 2019, concluding that immediate removal of the focus tooth is the best approach to achieve the lowest LOS [17].

According to this study, a psychiatric disorder or dementia (19.9%) can be observed as a significant comorbidity in patients suffering from DNIs. In 2009, Daramola et al. reported a 10.4% rate for psychiatric illnesses [18]. In this subgroup, the majority of infections (54.5%) were odontogenic. This might be due to poor commitment to dental hygiene. Kisely et al. 2016, demonstrated an association between poor oral health and psychiatric morbidity in a meta-analysis of 334,503 patients [19]. Similarly, dementia has been strongly linked to oral health problems [20]. Diabetes is a well described risk factor for DNIs [5, 11, 18]. In the present study, we confirmed that the median overall LOS (6 days) was more often exceeded by diabetic patients and significantly more ICU treatment was needed. These diabetic patients were more likely to need repeated surgery, although the complication rate was not substantially different. Furthermore, all somatic comorbidities (excluding psychiatric and dementia) had an association with complications and the need for repeated surgery.

Gas formation has traditionally been a sign of fulminant and rapidly progressive infections such as necrotizing fasciitis, which is present in 56.39% of the cases of cervical NF in recent meta-analysis [21, 22]. Moreover, with odontogenic infections, crepitus was described as a sign of severity by Alotaibi et al. 2015 [23]. In our study, 15 (83.3%) of the NF cases presented gas formation as well as 10.2% (*n* = 26) of the DNIs others than NF. We confirmed that gas formation was linked to a more severe course with a higher complication rate, increased mortality, longer hospitalization and both increased ICU treatment and a longer ICU stay; this was also true for DNIs not diagnosed as NF. Lin et al. 2014, stated that gas formation detected on CT scans of DNIs was a sign of anaerobic pathogens and a higher complication rate [24]. Because of the retrospective nature of our study, it is possible that some cases of true NFs were miscategorized using a strict criteria of necrosis during surgery and although that might increase the difference, it is unlikely to change all the results indicating a more severe course.

DNIs continue to cause significant morbidity and mortality. We found 22% suffering complications and 1.4% mortality. Several other authors have reported similar statistics, such as 18% had complications and a mortality rate 0.3% by Boscolo-Rizzo 2012, 16.2% and 1.6% by Huang 2004, 27.5% and 2.6% by Ridder 2005 [5, 11, 14]. Nevertheless, the differences in complications reported are present and should be noted. In this study, it is probable that excluding conservatively treated DNIs and including necrotizing fasciitis, we have created a bias towards analyzing infections with a more severe course. The benefit of HBO treatment as an adjuvant in cervical necrotizing infections is unsolved [25–29]. Descending mediastinitis can still be considered a highly life-threatening condition having a reported mortality rate varying from 0 to 40%, being overall 17.5% in the latest systematic review of 84 patients by Prado-Calleros 2016 [5, 22, 30–33]. In the present series 11 out of 12 mediastinitis patients survived, resulting in an 8.3% mortality rate. HBO therapy was utilized in nine cases (75%) and was started immediately (at least within 24 h) after surgery in most cases (*n* = 7/9). Shaw et al. 2014, concluded that HBO treatment can be beneficial especially for those patients who were very ill with necrotizing soft tissue infections [29]. HBO treatment could be beneficial with the most severe cases of DNIs, such as mediastinitis, when considering our results of mediastinitis survival, although a need for a larger and case-controlled series is evident.

We found *Streptococcus species* in 63.7% of the cases and these have been widely reported in earlier studies [5, 7, 8, 11, 14]. The Streptococcus anginosus group (35.8%) has been shown to be an important pathogen in the head and neck area causing locally extensive infections and a metastatic spread of infection [34]. Anaerobic *Prevotella species* were involved in one third (36.3%) of all cases. In 1994, Shinzato et al. showed in a mouse model that the Streptococcus anginosus (former milleri) group in synergy with *Prevotella intermedia* produced a more severe pneumonia and increased mortality [35]. This synergy is possibly present in the pathogenesis of DNIs and should be further examined. Possible changes in the antibiotic susceptibility could contribute to more severe course of infection, although the specific analysis on sensitivities was lacking. In odontogenic infections, sensitivity rate for penicillin is reported high (87.1–100%) for these viridans streptococcal species [36, 37]. Nevertheless, bacteria presenting low susceptibility to one or more of the standard antibiotic therapy regimes, have been shown to cause spreading infections and increased need and longer stay for inpatient treatment [36–38].

According to the present evaluation the incidence of DNIs is not decreasing. The annual numbers have risen from 14 to 24 cases per year. Odontogenic etiology was the predominant cause of these infections in this study and we demonstrated a clear increase compared to previous decades. Seppänen et al. 2010 found similar upwards trend in Helsinki, Finland, Thomas et al. 2008 in UK national data, and Bottin 2003 in Italy [9, 10, 39]. Patients were significantly older in the present study which could be a result of the ageing population in general. Age has been shown to be a risk factor for DNIs [7, 40]. Moreover, considering the aging population, more patients are having full dentition until the last years of life. Fu et al. 2018 stated that admissions to ICU for DNIs were rising [41]. We found a rising tendency between two cohorts (20.8–24.3%) but the difference did not reach statistical significance. Seppänen et al. 2010 stated odontogenic maxillofacial infections had become more severe over a 10-year period and patients with underlying diseases had increased in their latest cohort [9]. Comorbidities were significantly increased in our recent data. The results could be due to differences in noted comorbidities but nevertheless patients were significantly older and potentially more morbid. Furthermore, the complication rate was significantly higher in the present series. One reason for the higher percentage might be the wider scale of complications noted from the patient data. Mortality was increased from 0 to 1.7% because of four lethal cases occurring during the latest decade. Increasing incidence and intermittent probability might partially explain that rise, but finding the causes of growing mortality should trigger further studies. All these trends highlight the growing complexity of DNIs and should emphasize further development of efficient management and treatment protocols.

The most common etiologies were odontogenic and pharyngotonsillar in the present and comparative cohorts although the proportion had significantly increased in the recent group. This finding is in concordance with earlier reports [5, 7, 10]. The unknown etiologies were reduced in the latest data and this might be due to the strict diagnostics or a more detailed data collection. An OPG was obtained (53.1%) to identify dental foci, which is more frequent compared to previous reports from Staffieri 2014 (36.5%) and Bottin 2003 (43%) [10, 42]. In the odontogenic subgroup an OPG was available in 79.7% of the cases. In the remaining 20.3%, dental imaging was possibly obtained from nonelectric versions in earlier visits by or information from CT scans was used. As earlier reported by several writers parapharyngeal, submandibular and retropharyngeal spaces were those most commonly involved in both groups [5, 7, 10]. Although multilocular involvement was not mentioned in the previous data which can have affected the exact proportions.

The limitations of the present study are acknowledged and mostly due to the retrospective nature of the analysis. The lack of similar detailed data from previous decades weakened the analysis in this respect.

## Conclusion

We found a clear upward trend in the annual numbers of DNIs. Oral health problems, and furthermore, odontogenic infections are a growing challenge. Along with prevention, the early removal of an affected tooth or teeth is essential to achieve the lowest hospital stay when managing these advanced infections. DNIs continue to cause lethal complications and aggravating comorbidities need to be taken into account. Gas formation is predictive of a more severe course of infection indicating an increasing mortality, a need for multidisciplinary treatment at the ICU and a longer hospital stay. HBO therapy as an adjuvant might be beneficial in most severe cases such as descending mediastinitis. Early recognition and prompt management are crucial to prevent sequelae.
